# Quantitative measurements of reactive oxygen species partitioning in electron transfer flavoenzyme magnetic field sensing

**DOI:** 10.3389/fphys.2024.1348395

**Published:** 2024-02-02

**Authors:** Chase K. Austvold, Stephen M. Keable, Maria Procopio, Robert J. Usselman

**Affiliations:** ^1^ Chemistry and Biochemistry, Montana State University, Bozeman, MT, United States; ^2^ Molecular Biophysics and Integrated Bioimaging Division, Lawrence Berkeley National Laboratory, Berkeley, CA, United States; ^3^ Biophysics, Johns Hopkins University, Baltimore, MD, United States; ^4^ Chemistry and Chemical Engineering, Florida Institute of Technology, Melbourne, FL, United States; ^5^ Computational Research At Florida Tech, Melbourne, FL, United States

**Keywords:** radical pair mechanism, flavoenzymes, reactive oxygen species, quantum biology, magnetic field effects, mitochondria, bioenergetics

## Abstract

Biological magnetic field sensing that gives rise to physiological responses is of considerable importance in quantum biology. The radical pair mechanism (RPM) is a fundamental quantum process that can explain some of the observed biological magnetic effects. In magnetically sensitive radical pair (RP) reactions, coherent spin dynamics between singlet and triplet pairs are modulated by weak magnetic fields. The resulting singlet and triplet reaction products lead to distinct biological signaling channels and cellular outcomes. A prevalent RP in biology is between flavin semiquinone and superoxide (O_2_^•−^) in the biological activation of molecular oxygen. This RP can result in a partitioning of reactive oxygen species (ROS) products to form either O_2_^•−^ or hydrogen peroxide (H_2_O_2_). Here, we examine magnetic sensing of recombinant human electron transfer flavoenzyme (ETF) reoxidation by selectively measuring O_2_^•−^ and H_2_O_2_ product distributions. ROS partitioning was observed between two static magnetic fields at 20 nT and 50 μT, with a 13% decrease in H_2_O_2_ singlet products and a 10% increase in O_2_^•−^ triplet products relative to 50 µT. RPM product yields were calculated for a realistic flavin/superoxide RP across the range of static magnetic fields, in agreement with experimental results. For a triplet born RP, the RPM also predicts about three times more O_2_^•−^ than H_2_O_2_, with experimental results exhibiting about four time more O_2_^•−^ produced by ETF. The method presented here illustrates the potential of a novel magnetic flavoprotein biological sensor that is directly linked to mitochondria bioenergetics and can be used as a target to study cell physiology.

## 1 Introduction

The interaction between living systems and magnetic fields has recently witnessed a renewed interest due to the importance of possible quantum processes harnessed by living systems (Lambert et al., 2013; Kim et al., 2021). For utility in biological applications, a better understanding of the quantum mechanisms at the biomolecular level is needed to direct desired outcomes in cell physiology (Usselman et al., 2014; Usselman et al., 2016; Franco-Obregón, 2023). Among the proposed mechanisms for weak magnetic field sensing in biology (Qin et al., 2016; Nordmann et al., 2017; Lin et al., 2019; Gao et al., 2021), the leading quantum process is the radical pair mechanism (RPM). Living systems are replete with forming and breaking chemical bonds, with many reactions creating radical pair (RP) intermediates. However, biological RP reactions must satisfy specific physical and chemical requirements to accomplish magnetic sensing (Timmel et al., 1998; Player and Hore, 2019). The flavoprotein cryptochrome has been proposed to be a biological magnetic receptor (Ritz et al., 2000), where a RP is initialized by either photoexcitation or during the redox cycle of the flavin cofactor (Maeda et al., 2008; Hogben et al., 2009; Hore and Mouritsen, 2016). Other protein systems have been suggested to sense weak magnetic fields (Jones, 2016). Here, we demonstrate a general method, based on product yield detected magnetic resonance (PYDMR)^1^, to investigate the RP-based magnetic sensing in reduced flavoenzymes that produce reactive oxygen species (ROS). This method is complementary to photoexcitation measurements, such as (auto)fluorescence in RP reactions (Evans et al., 2015; Ikeya and Woodward, 2021), and provides additional information via quantitative measurements on ROS product yields.

ROS are products of oxygen-dependent life in aerobic metabolism and are generally derived from molecular oxygen (O_2_) in redox active processes (Jones and Sies, 2015). The main initial ROS products in metabolism are superoxide (O_2_^•−^) and hydrogen peroxide (H_2_O_2_) (Schieber and Chandel, 2014), which have inherent chemical properties that coincide with their reactivity and regulation within biological pathways. Under normal physiological levels, ROS serve as oxidative signaling molecules that affect biological and physiological processes, where excessive ROS levels lead to oxidative stress (Sies, 2017). Excessive oxidative stress can result in damage to lipids, proteins, and DNA within cells and has been linked the onset of several diseases (Cross et al., 1987). Cells utilize ROS in key signal transduction mechanisms and mitochondria bioenergetics that are crucial for adaptation to a changing oxidative environment (Wood et al., 2003; Brandes et al., 2009).

Mitochondria are the major source of ROS, with topological assays that show ROS production and contributions from different metabolic sites (Brand, 2010; Dröse and Brandt, 2012). β-oxidation is a primary catabolic pathway that involves the degradation of saturated fatty acids and has been shown as a source of ROS formation (Bartlett and Eaton, 2004; Rosca et al., 2012). Electron transfer flavoenzyme (ETF) is the main electron acceptor in mammalian β-oxidation and serves as an electron funnel from at least 11 unique flavoprotein dehydrogenases and some amino acid catabolism (Roberts et al., 1996). The electrons are then transferred to the ubiquinone pool (Q-pool) via the inter-membrane bound electron flavoprotein ubiquinone oxidoreductase (ETF-QO) (Watmough and Frerman, 2010). ETF shuttles electrons by a single flavin adenine dinucleotide (FAD) cofactor. In addition to electron transfer, the ETF FAD site can serve as a secondary role for a ROS oxidative signaling terminal point, which involves the partitioning of O_2_^•−^ and H_2_O_2_. ROS are produced through the interaction of the reduced flavin cofactor with molecular oxygen, presumably because of a disruption of electron flow to the Q-pool (Olsen et al., 2007; Burke, 2023).

The local flavin protein environment tunes the relative thermodynamic midpoint potentials for the three flavin redox states of oxidized quinone (0e^−^), radical semiquinone (1e^−^), and fully reduced hydroquinone (2e^−^) (Romero et al., 2018). Therefore, flavoenzymes produce exclusively O_2_^•-^ (1e^−^) or H_2_O_2_ (2e^−^) or populations of both ROS, depending on the local flavin environment. For example, flavodoxins are 1e^−^ transferases because of the high flavin redox couple, and alternatively, dehydrogenases form mainly H_2_O_2_ due to the low flavin redox couple. For a magnetic field sensitive flavoenzyme, the redox couple must be sufficiently low, but not too high, to produce both O_2_^•−^ and H_2_O_2_. Human ETF midpoint potentials have been determined for the Fl_hydroquinone_/Fl_semiquinone_ (−75 mV) and for the Fl_semiquinone_/Fl_quinone_ (+15 mV), with human ETF shown to produce both O_2_^•−^ and H_2_O_2_ (Rodrigues and Gomes, 2012; Henriques et al., 2021). Some of the local protein environment factors the modulate redox potentials include solvent accessibility, hydrogen bonding, backbone amide dipoles, and local charge (Swanson et al., 2008; Usselman et al., 2008).

**SCHEME 1:**
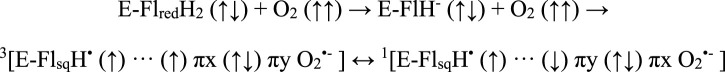
A proton coupled electron transfer activates molecular oxygen to form a triplet born spin correlated radical pair, with singlet and triplet coherent dynamics affected by magnetic fields.

**FIGURE 1:**
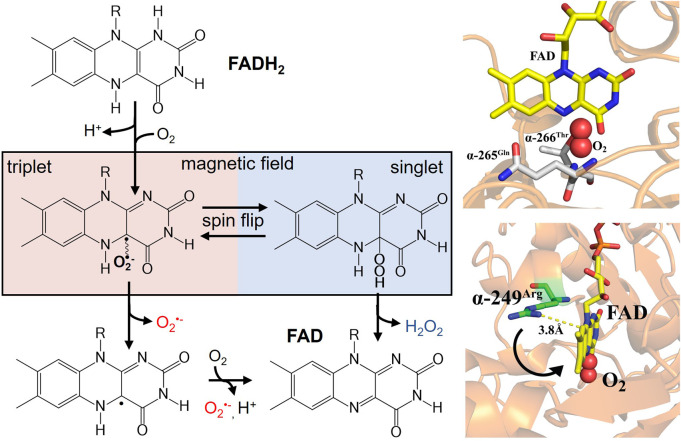
(left) Activation of molecular oxygen by reduced flavin to produce a spin-correlated radical pair between flavin semiquinone and O_2_^•−^. A magnetic field sensitive divergent point exists for oxidative signaling that can produce either O_2_^•−^ (triplet product) or H_2_O_2_ (singlet product). Right (bottom) ETF X-ray crystal structure (PDB ID: 1EFV) of FAD cofactor in close proximity to the proposed semiquinone stabilizing residue α-249Arg. The distance indicates a conformational movement is needed for stabilization of the radical pair. Molecular oxygen is modeled into the proposed binding site nested between conserved hydrogen bonding partners (right top).

Redox active flavoproteins can undergo a proton coupled electron transfer (1e^−^) to activate O_2_ to create a caged RP between the flavin semiquinone (FADH^•^) and O_2_^•−^ anion, Scheme 1 (Bruice, 1984; Massey, 1994; Reece et al., 2006; Chaiyen et al., 2012; Gadda, 2012; Imlay, 2013). Because the ground state of O_2_ is a triplet state, FADH^•^:O_2_^•−^ is initially created in the triplet state. The FADH^•^:O_2_^•−^ presents a spin selective divergent point to release specific ROS products, where the reaction can either release O_2_^•−^ through the triplet product channel or with an additional electron transfer can release H_2_O_2_ through the singlet channel, Figure 1.

At the RP formation, applied magnetic fields and local hyperfine interactions affect spin coherences that mix between the triplet and singlet states (Schulten and Wolynes, 1978). Therefore, internal and external magnetic fields can impact ROS products, redistributing the relative product ratios. Manipulating ROS levels using magnetic fields can potentially function as a cellular “redox switch,” which could have significant biological effects. Further understanding of quantum processes in this RP redox system could elucidate fundamental knowledge in ROS quantum biology.

To better understand the role of the RPM in ROS production at the biomolecular level in flavoenzymes, we selectively measured O_2_^•−^ and H_2_O_2_ products with the static magnetic field artificially set to 50 µT (Earth’s magnetic field) and 20 nT static magnetic fields for recombinant human ETF. The methodology presented here can be used to study magnetic field effects in flavoproteins that are potential candidates for magnetic biosensors.

## 2 Materials and methods

### 2.1 Recombinant human ETF ROS assays

The growth and purification of human ETF was completed by adopting a procedure as previously described (Roberts et al., 1995; Austvold, 2019). Flavin loading in ETF was determined to be 97% by protein and flavin absorbance at A_280_ and A_450_, respectively. A 24 µM solution of ETF was prepared by diluting a stock solution in 10 mM Tris buffer at pH = 7.5. The ETF solution was then transferred into an anaerobic cuvette and purged with Argon gas for 20 cycles. Reduced ETF was formed by enzymatic reduction with catalytic concentrations of medium chain acyl-coenzyme A dehydrogenase (MCAD) and octanoyl-CoA. An anerobic solution of MCAD and octanoyl-CoA was added to initialized reduction with final concentrations of 20 µM ETF, 0.02 µM MCAD, and 100 µM octanoyl-CoA. The reaction was monitored at flavin A_450_ until the spectrum remained unchanged, approximately 15 minutes at 20°C. Selective ROS assays were used to quantify H_2_O_2_ and O_2_^•−^ upon the re-oxidation of ETF reduced FAD cofactor. 20 μM ETF at 250 µL of the enzymatically reduced protein was maintained in an anaerobic environment, then O_2_ was introduced to the system by the addition of oxygenated 250 µL Tris buffer pH 7.5 (∼250 µM O_2_) containing the reagents for separate ETF ROS assays. Amplex Red (100 μM, 0.4 U/mL HRP) and dihydroethidium (DHE, 50 µM) were used to selectively measure H_2_O_2_ and O_2_^•−^, respectively, with reoxidation occurring within 10 min by monitoring A_450_. Four separate samples were analyzed and conducted in triplicates. One ETF sample for each ROS assay and their corresponding blanks, with quantitation determined by standard curves for each ROS assay.

### 2.2 Magnetic field Instrumentation

A tri-axial Helmholtz coil system with a 6-channel DC power supply was used to control the static magnetic field strength and direction in each of two temperature controlled environments (Usselman et al., 2014). A triaxial magnetic field sensor provided automatic feedback (PID) that allowed for real-time control of magnetic fields to cancel out other static magnetic fields present. The experiments were carried out in a Faraday cage. The static magnetic fields were set to either 50 µT or 20 nT perpendicular to the standing cuvette. The samples were held at 20°C during the re-oxidation of ETF for the selective ROS assays.

### 2.3 Modeling of realistic flavin-superoxide radical-pair reactions under static magnetic fields

**FIGURE 2:**
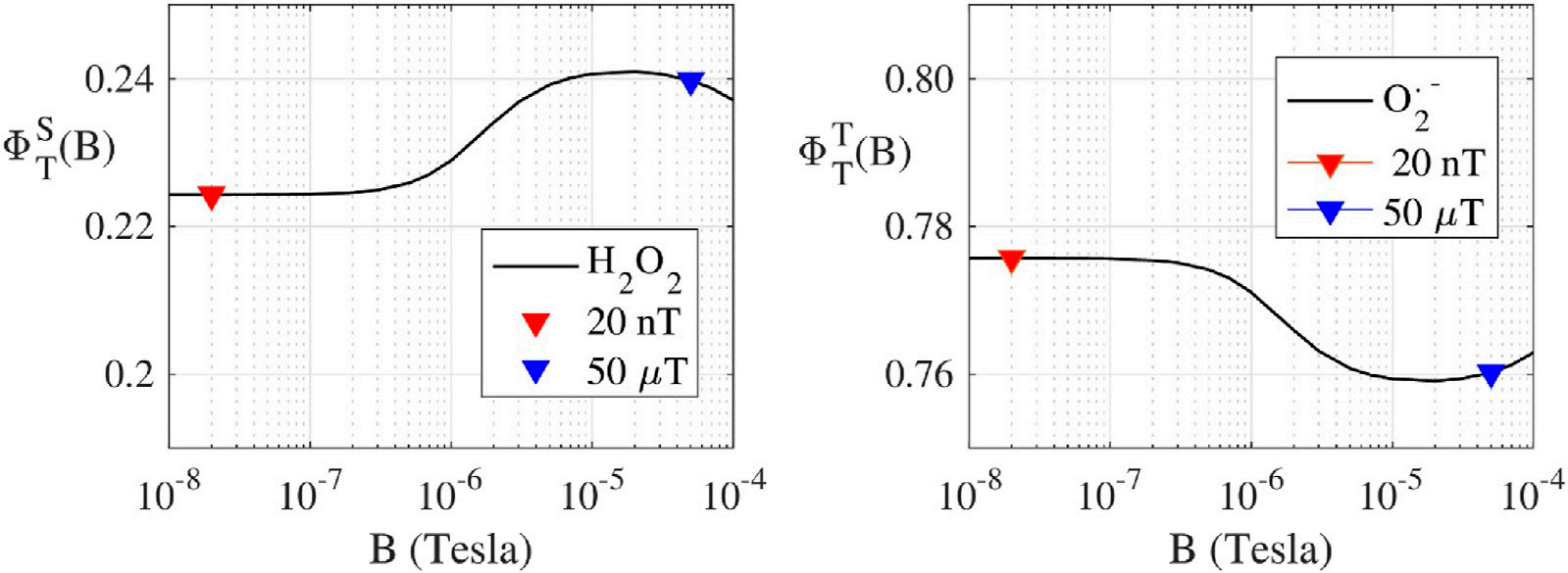
(Protein bound-Suproxide) Spin dynamic simulation of the singlet yield $\phi_{S}^{T}$ (top H_2_O_2_ production) and triplet yield $\phi_{T}^{T}$ , (O_2_^•−^ production) of a triplet born radical-pair, as a function of the external magnetic field B (in log scale). The radical-pair model includes 7 isotropic hyperfine interactions in the flavin radical, and one isotropic hyperfine in the superoxide radical. The radical-pair lifetime was set to 10 μs. The red triangle represents values at 20 nT, and the yellow triangle values at 50 μT.

Following the RP-based magnetoreception theory (Schulten and Wolynes, 1978; Timmel et al., 1998; Ritz et al., 2000; Procopio and Ritz, 2016), we have calculated the singlet ( $\phi_{S}^{T}$ ) H_2_O_2_ and triplet ( $\phi_{T}^{T}$ ) O_2_^•-^ product yields of a triplet born flavin-superoxide RP model as a function of the external magnetic field. The lifetime of the RP was set to 10 µs, and we have assumed that spin relaxation times are longer than the radical-pair lifetime. A theoretical static magnetic field dose-response curve, ranging from 10 nT to 100 μT, was calculated for ROS production. ROS product yields are shown in Figure 2 for the H_2_O_2_ production (left), and for the O_2_^•−^ production (right), where red triangles depict ROS production at 20 nT and blue triangles at 50 µT.

Our calculations implemented a realistic flavin-superoxide RP model, where O_2_^•−^ is considered bound to a protein cofactor. We chose this model because an unbound O_2_^•−^ would have a spin relaxation time too fast for magnetic field effects to occur (Player and Hore, 2019). In the bound case, O_2_^•−^would experience some hyperfine interactions from the solvent, which have been predicted to be up to 120 μT (Hogben, 2011). Thereby, we model a RP with one hyperfine interaction in the O_2_^•−^ radical, and we choose the first seven largest hyperfine interactions in the flavin radical (Lau et al., 2012). Furthermore, we have considered the hyperfine interactions to be isotropic because the two radicals tumble in solution, thus the anisotropy is averaged out.

## 3 Results

### 3.1 ROS partitioning assays

ROS partitioning experiments were conducted with 10 µM ETF and performed in triplicates for each magnetic field strength. The Amplex Red assay measured the amount of H_2_O_2_ produced within the reoxidation ETF reaction. The measured H_2_O_2_ average concentration for 20 nT was 2.1 ± 0.3 µM and for 50 µT the average concentration was 2.4 ± 0.2 µM. The results indicate a 13% decrease in H_2_O_2_ from 50 μT to 20 nT. The DHE O_2_^•-^ assay showed the amount of O_2_^•−^ produced during the re-oxidation of ETF for 20 nT was 10.6 ± 1.4 µM and for 50 µT was 9.6 ± 0.8 µM. The results show a 10% increase in O_2_^•−^ production with the decrease in magnetic field strength from 50 μT to 20 nT. Relative ROS yields show an increase from four to five times more O_2_^•−^ produced than H_2_O_2_ upon lowering the magnetic field, illustrating an increasing preference for the O_2_^•−^ triplet channel product. Comparative analysis between the two different magnetic fields has *p*-values for O_2_^•−^ and H_2_O_2_ triplicate experiments of 0.29 and 0.36, respectively. Both values indicate a non-significant difference for experiments in each field condition, exemplifying the need to reduce error in ROS flavoprotein assays. Theoretical calculations for H_2_O_2_ production decreases from 50 µT (0.240) to 20 nT (0.220) of about 0.016 (7% decrease). Conversely O_2_^•−^ production increases of the same amount ( $\phi_{S}^{T}+$ $\phi_{T}^{T}$ = 1). Both results are in agreement with the measured ROS products levels.

## 4 Discussion

Under normal physiological conditions, ROS are oxidative signaling molecules that regulate a cellular redox network (Sies et al., 2022). Overproduction of ROS can lead to oxidative damage and a host of physiological or pathological outcomes. To better understand the phenotypic boundary between oxidative signaling or stress, biomolecular ROS quantification is essential. We demonstrate an experimental approach that can be utilized for quantitative measurement of flavoenzyme ROS generation. ETF was chosen due to its central role in bioenergetics and electron transfer pathway that feeds electrons to the mitochondria Q-pool. ETF was enzymatically reduced by MCAD and then ROS was selectively measured upon the reoxidation of the flavin cofactor. Our goals were to measure the relative proportions of ROS and the impact of magnet sensing on ROS product distributions of ETF. The reoxidation of ETF produced ROS partitioning of approximately four-fold more O_2_^•-^ than H_2_O_2_, different than other findings of ETF ROS production (Rodrigues and Gomes, 2012). Flavin thermodynamic redox couples contribute to the observed ROS partitioning, in which the local protein environment tunes the flavoprotein redox properties (Swanson et al., 2008; Usselman et al., 2008; McDonald et al., 2011; Gran-Scheuch et al., 2023).

In addition, protein-protein interactions, such as with MCAD, induce conformational changes that can also impact midpoint potentials, analogous to points mutations in the vicinity of the flavin cofactor (Swanson et al., 2008; Usselman et al., 2008; Rodrigues and Gomes, 2012). Given that the amino acid environment in proximity to the flavin primarily determines the thermodynamic midpoint potentials, the local environment ultimately dictates normal ROS products and distributions in flavoenzyme structure-function relationships. The peptide environment around the FAD cofactor is not only crucial for redox tuning, but also serves as a flexible site for electronic coupling during electron transfer. A highly conserved arginine residue near the FAD cofactor was proposed to be responsible for stabilizing the superoxide radical pair is (Figure 1 right-bottom) (Roberts et al., 1996). Distance measurements indicate the need for a conformational movement of this residue during electron transfer to stabilize the semiquinone state and allow for ROS partitioning.

**FIGURE 3:**
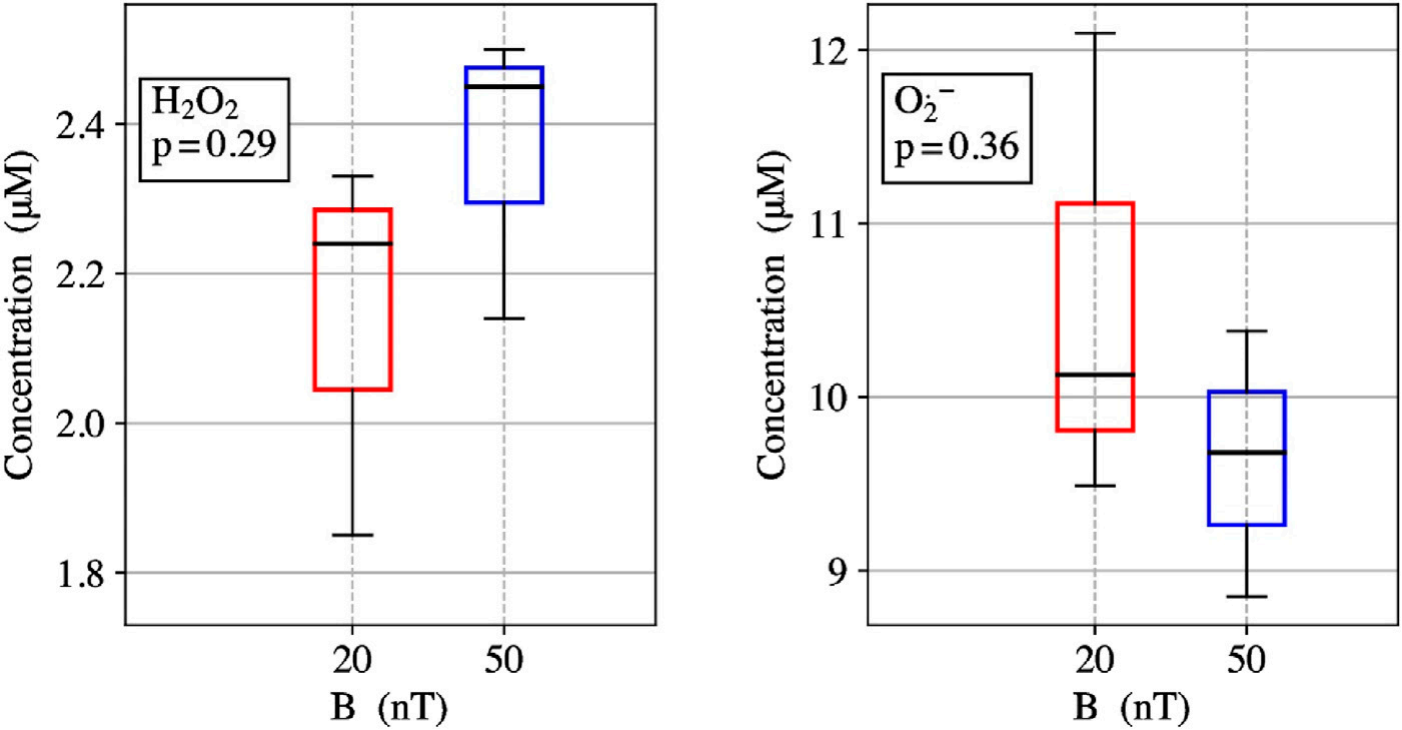
(left) ETF reoxidation for measure concentrations of H_2_O_2_ singlet product yields at 20 nT and 50 µT. (right) ETF reoxidation for measure concentrations of O_2_^•−^- triplet product yields at 20 nT and 50 µT.

The protein molecular determinants that give rise to magnetic sensing are not well-understood and are perhaps rare in biology (Messiha et al., 2015). We proposed ETF as a potential redox magnetic sensor (Austvold, 2019), where flavin semiquinone radical and O_2_^•−^ form a spin correlated RP initialized in the triplet-state, leading to characteristic magnetic field dependence on ROS product yields. Here, experimental results were conducted to compare the Earth’s static magnetic field at 50 µT and a lower static magnetic field at 20 nT. ETF reoxidation assays measured a 13% decrease in H_2_O_2_ production and an increase of 10% O_2_^•−^ from 50 µT to 20 nT, Figure 3. The experimental ROS partitioning demonstrates one of the hallmark quantum signatures of the RPM in operation for ETF. Of critical importance is the spatial arrangement of O_2_ relative to the flavin group (Chaiyen et al., 2012), as well as the required binding time of O_2_^•−^ in proximity to the semiquinone for sufficient spin correlation. Recent molecular dynamics simulations discovered several novel ETF oxygen binding sites in ETF (Nielsen et al., 2019; Salerno et al., 2022), suggesting that ETF can activate O_2_ through perhaps an outer sphere electron transfer. The RP distance can affect spin relaxation and thus magnetic sensing in the radicals, whereas the problems with spin relaxation can be essentially removed by a radical scavenger by the quantum Zeno effect (Kattnig, 2017).

Using the RP theory avian magnetoreception (Ritz et al., 2000), simulations have been performed to quantify ROS products that are dictated by coherent dynamics of singlet and triplet RP spin states (Procopio and Ritz, 2016). We have determined singlet and triplet product yields, and thus relative distributions of O_2_^•−^ and H_2_O_2_ as a function of the static magnetic field strength. A realistic model was used to calculate the ROS products yields for static magnetic fields ranging from 10 nT to 100 μT. The theoretical RP results correlate with the observed ROS yields of a decrease in H_2_O_2_ singlet products and an increase in O_2_^•−^ triplet products from 50 μT to 20 nT for the reoxidation of ETF, Figure 2. Thus, controlling ROS product channeling can be accomplished by using specific magnetic fields and configurations (Franco-Obregón, 2023).

Over the past 2 decades, cryptochrome experiments have shown increasing evidence for magnetic sensing, and more generally, the involvement of ROS (Ritz et al., 2000; Solov’yov and Schulten, 2009; Martino and Castello, 2011; Muller and Ahmad, 2011; Arthaut et al., 2017; Pooam et al., 2020). We previously reported that flavin-superoxide RP could be a broader magnetic sensing system in redox cell biology (Usselman et al., 2014; Usselman et al., 2016). Our ROS cellular research, combined with flavin-superoxide RP theoretical models, supports biomolecular ROS distributions from the results obtained through the re-oxidation of ETF. However, recently the primary magnetic receptor was suggested to be O_2_^•−^ itself and perhaps O_2_^•−^ dismutation, with observations supported by cellular (Martino and Castello, 2011), mouse (Carter et al., 2020) and planarian models (Van Huizen et al., 2019; Kinsey et al., 2023). If flavin-superoxide RP magnetic sensing is occurring, the discrepancy among the reports could involve magnetic field conditions that target different flavoenzymes, i.e., oxidases or monooxygenases (Massey, 1994; Imlay, 2013; Usselman et al., 2014; Gran-Scheuch et al., 2023). In addition, less is known about the initial adaptive ROS cellular responses because of the intrinsic antioxidant regulatory systems (Sies et al., 2022). Nonetheless, targeting different ROS producing systems greatly offers an expanded approach for magnetic field intervention (Vecheck et al., 2024) to remotely hack the redox code (Jones and Sies, 2015) and impart select cellular physiological responses.

### 4.1 Limitations

One of the major challenges in studying ROS in biological systems is the difficulty of measurement and quantitation (Dikalov et al., 2007; Kalyanaraman et al., 2014). Moreover, ROS are not only highly transient but are produced by many different systems in cell physiology (Murphy et al., 2022), whereas recombinant flavoproteins offer a reductionist biomolecular approach to identify magnetic-induced ROS partitioning. However, uncertainty in protein concentration and flavin loading can lead to errors as well, in addition to the ROS assays, especially measuring superoxide. In addition, changes in reaction yields via the RPM are usually less than 10%, therefore, requiring an increased minimization of error in experimental procedures.

## 5 Conclusion

Many oxidative metabolic pathways occur within the mitochondria and involve redox intermediates that can interact with O_2_ to produce ROS, including ETF/ETF-QO (Watmough and Frerman, 2010; Perevoshchikova et al., 2013). Therefore, mitochondria are a vital source of ROS production within eukaryotic cells (Jones and Sies, 2015) and throughout the microbial biosphere (Imlay, 2013). ROS signaling by mitochondrial enzymes, including ETF, play a fundamental role in oxidative signaling (Sies et al., 2022), where the progression to cellular dysfunction can ultimately lead to inflammation and disease. While the effects of different magnetic field environments can alter ROS production (Barnes and Greenebaum, 2018; Gurhan et al., 2021), the persistent changes of oxidative signaling can have longer term impacts on cell physiology (Thoni et al., 2022; Franco-Obregón, 2023). Here, we show that ETF should be considered a target for further RPM investigations due to the importance of mitochondria bioenergetics, especially for biomedical engineering and therapeutic potential. Particularly, the intersection of electric voltages and magnetic spins offers a novel approach to investigate the connection between energy and living systems (Lee et al., 2023). The low magnetic fields strengths studied here also illustrate the importance of understanding how spin mechanisms could impact space health and agriculture.

## Footnotes

1 Also known as reaction yield detected magnetic resonance (RYDMR).
